# Emergency Valve-in-Valve Transcatheter Aortic Valve Implantation in a Patient With a Degenerated Surgical Xenograft and High Body Mass Index Presenting With Recurrent Pulseless Electrical Activity Arrest

**DOI:** 10.7759/cureus.111457

**Published:** 2026-06-24

**Authors:** Zanobia Sameer

**Affiliations:** 1 Cardiology, Basingstoke and North Hampshire Hospital, Basingstoke, GBR

**Keywords:** balloon aortic valvopathy, bioprosthetic valve degeneration, cardiac arrest, coronary protection, high bmi patient, multidisciplinary management, structural heart disease, valve-in-valve transcatheter aortic valve implantation (viv-tavi), vascular complication

## Abstract

Degeneration of bioprosthetic aortic valves is a recognised late complication that may present with severe stenosis, regurgitation, or acute haemodynamic compromise. Redo surgical aortic valve replacement in patients with obesity and multiple comorbidities carries substantial perioperative risk, making valve-in-valve transcatheter aortic valve implantation (ViV-TAVI) an important alternative. We report a middle-aged man with obesity and a prior bovine xenograft aortic valve replacement who presented with progressive heart failure, severe prosthetic aortic stenosis, and recurrent pulseless electrical activity arrest. He was stabilised with vasopressor support and emergency balloon aortic valvuloplasty before undergoing successful left transfemoral ViV-TAVI with coronary protection and common femoral artery repair. His postoperative course was complicated by delayed vascular infection and bleeding, requiring further surgical and endovascular intervention. This case highlights the role of urgent ViV-TAVI in critically unwell patients, the technical challenges of reintervention in obesity, and the importance of multidisciplinary management in complex structural heart disease.

## Introduction

Bioprosthetic aortic valves are commonly used due to their favourable haemodynamic profile and avoidance of long-term anticoagulation; however, they are inherently susceptible to structural valve degeneration (SVD).

Bioprosthetic aortic valves are prone to SVD over time, with progressive stenosis, regurgitation, or mixed dysfunction leading to recurrent heart failure symptoms and, in severe cases, haemodynamic collapse [1]. Valve-in-valve transcatheter aortic valve implantation (ViV-TAVI) has become an established alternative to redo surgical valve replacement in patients with degenerated surgical bioprostheses who are at high operative risk [2]. The technique involves the deployment of a transcatheter valve within the failed prosthesis, restoring valve function while avoiding repeat sternotomy. However, ViV-TAVI carries specific procedural risks, most notably coronary obstruction due to the displacement of bioprosthetic leaflets toward the coronary ostia. Coronary protection strategies, including guidewire placement with or without pre-positioned stents, are therefore employed in selected high-risk cases to reduce this risk. Coronary protection is an established strategy in valve-in-valve procedures when there is a risk of coronary obstruction [3].

Obesity adds procedural complexity to transcatheter valve therapy, particularly by increasing the likelihood of vascular access difficulty and access-site complications [4]. At the same time, outcomes in obese patients undergoing TAVI are often comparable to, and in some cohorts better than, those in normal-weight patients, supporting the so-called obesity paradox [5,6].

We report a complex case of a degenerated bioprosthetic aortic valve presenting with recurrent pulseless electrical activity arrest, requiring a staged rescue approach with initial stabilisation followed by definitive ViV-TAVI with coronary protection. This case highlights the challenges of managing acute structural valve failure and underscores the importance of anticipatory planning in high-risk valve-in-valve interventions.

## Case presentation

A 69-year-old man, fairly mobile and independent, with a history of surgical aortic valve replacement using a bovine xenograft seven years ago and atrial fibrillation treated with apixaban presented with progressive heart failure symptoms. He had gradually worsening breathlessness, paroxysmal nocturnal dyspnoea, and reduced functional capacity over several weeks. His clinical course was complicated by obesity with a body mass index (BMI) of 33.72 kg/m^2^ (weight: 116.8 kg; height: 177 cm^2^) which increased the technical complexity of both evaluation and subsequent intervention. Additionally, his past medical history included hyperlipidaemia, type 2 diabetes mellitus, alcoholic liver disease, and essential hypertension.

Earlier this year, he was admitted with shortness of breath and paroxysmal nocturnal dyspnoea, and his B-type natriuretic peptide level was markedly elevated at 2916 ng/L. He was found to be in atrial fibrillation and was treated with an intravenous furosemide infusion totalling 240 mg. His admission was prolonged by gout. His uric acid was 1221 umol/L, and he was treated with colchicine followed by allopurinol. He was eventually discharged after three weeks.

Subsequently, on the second admission, he suffered multiple pulseless electrical activity arrests. He was resuscitated with intravenous fluids. His initial blood pressure was 85 systolic but dropped to 70 systolic. He was resuscitated with dopamine and metaraminol and was transferred to intensive care for further support.

**Figure 1:**
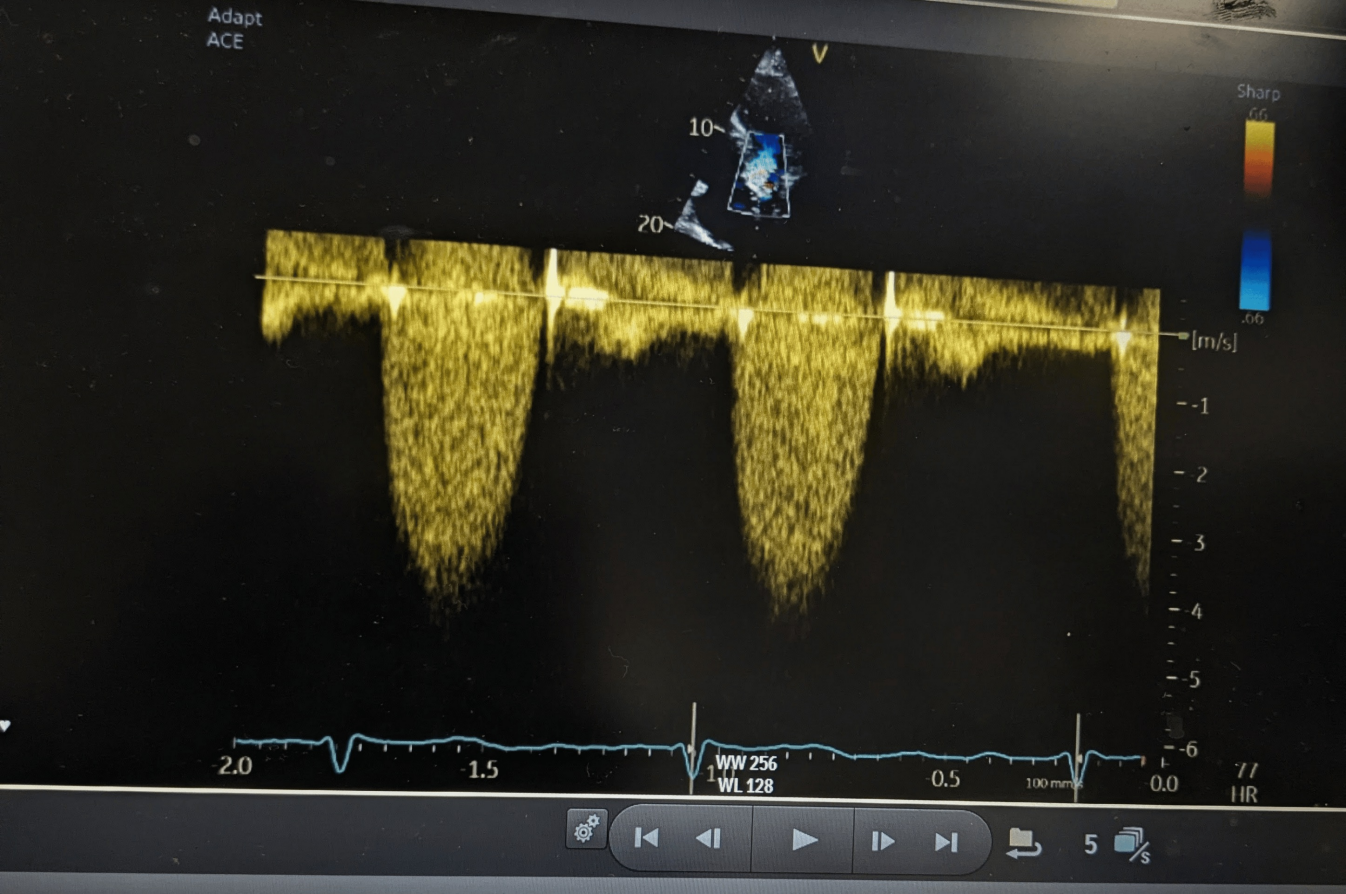
Continuous wave Doppler at the aortic area showing a peak velocity of 4.0 m/s

**Figure 2:**
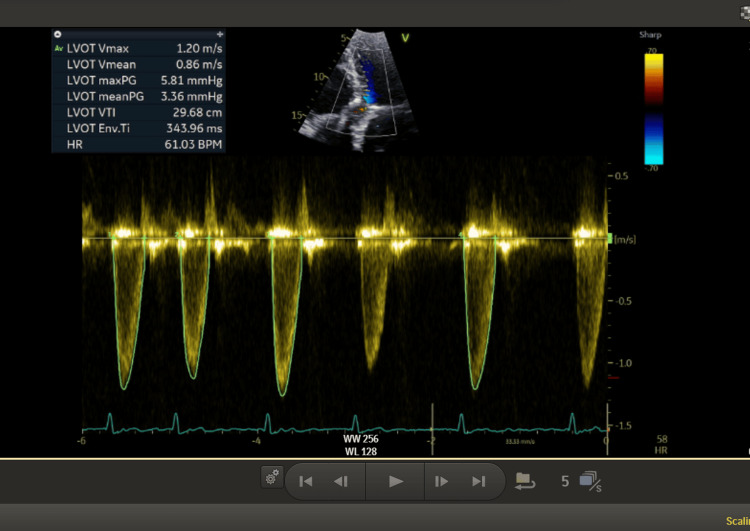
Continuous wave Doppler at the aortic area showing an aortic valve peak velocity of around 2.0 m/s

The transthoracic echocardiography during admission showed the following: significant calcification and degeneration of the tissue aortic valve replacement with critical stenosis with a mean gradient of 67 mmHg and a peak gradient of 121 mmHg, an aortic valve area of 0.55 cm^2^, mid-trans-valvular aortic regurgitation, mild left ventricular dilation, septal hypertrophy, visually estimated ejection fraction of 30-35%, posterior mitral annular calcification with fixed posterior leaflet, mild mitral stenosis and mitral regurgitation, normal right ventricular size with function, and normal left atrium. Figures 1-2 show a pre- and post-TAVI continuous wave Doppler at the aortic area, respectively.

There were no rhythm strips from the first two events of pulseless electrical activity arrests, but there was a rhythm strip from the last event showing a period of slow atrial fibrillation down to 30 bpm. The QRS was narrow, and the rate was irregularly irregular, which goes against complete heart block. Throughout this time, his electrocardiogram (ECG) showed rate-controlled atrial fibrillation with rates in the region of 70-85 bpm, with no evidence of acute ischaemia.

Because of the critical haemodynamic instability and the severity of the prosthetic obstruction, emergency balloon aortic valvuloplasty was undertaken as a bridging measure. A temporary pacing wire and intra-aortic balloon pump were used to support haemodynamics during this period after discussion in a multidisciplinary meeting between valvular and interventionalist cardiologists.

Definitive treatment was done in the form of TAVI, where the patient underwent left transfemoral ViV-TAVI with a 26 mm Edwards SAPIEN 3 valve (Edwards Lifesciences, Irvine, California, United States). A pre-TAVI computed tomography (CT) aortogram, shown in Video 1, was performed to measure the size of the aorta. Coronary protection was performed with left main stenting because valve-in-valve procedures carry a recognised risk of coronary obstruction in selected anatomies [3,7]. The common femoral artery was surgically repaired after the procedure. The intervention resulted in successful valve deployment and initial haemodynamic stabilisation.

**Video 1:** Pre-transcatheter aortic valve implantation computed tomography aortogram

His post-procedural course was complicated by significant left groin bleeding and subsequent abscess formation within 1-2 months of the procedure. He required placement of a Viabahn stent (Gore Medical, Newark, Delaware, United States), followed by obturator bypass with stent removal and surgical flap coverage. A CT angiogram performed three months later, which is shown in Video 2, confirmed a patent bypass and a stable retroperitoneal haematoma, after which he was discharged.

**Video 2:** Computed tomography angiogram showing a haematoma and a stent

The patient's key clinical events, investigations, and interventions are summarised in Table 1.

**Table 1:** Timeline of clinical events

Timeline	Event	Intervention/findings
Seven years ago	Surgical aortic valve replacement	Bovine xenograft implanted
First admission	Heart failure admission	B-type natriuretic peptide level 2916 ng/L; atrial fibrillation; IV furosemide 240 mg
Second admission	Echocardiography	Severe prosthetic aortic stenosis; mean gradient 67 mmHg; peak gradient 121 mmHg
	Clinical deterioration	Multiple pulseless electrical activity arrests; intensive care unit admission
	Bridging procedure	Emergency balloon aortic valvuloplasty; temporary pacing; intra-aortic balloon pump
	Definitive intervention	Left transfemoral valve-in-valve transcatheter aortic valve implantation with a 26 mm Edwards SAPIEN 3 valve
	Procedural adjuncts	Coronary protection with left main stenting; common femoral artery repair
1-2 months after the procedure	Access-site complication	Groin bleeding and abscess formation
3 months after the procedure	Salvage treatment	Viabahn stent, obturator bypass, stent removal, flap coverage
3 months and 5 days after the procedure	Follow-up imaging	Patent bypass; stable retroperitoneal haematoma
3.5 months after the procedure	Outcome	Discharged

## Discussion

Structural degeneration of bioprosthetic aortic valves can lead to severe haemodynamic compromise, including cardiogenic shock and cardiac arrest [1]. In such settings, urgent intervention is necessary. ViV-TAVI is now widely accepted as an alternative to redo surgical aortic valve replacement for selected patients with failed surgical bioprostheses, particularly when surgical risk is high [2].

Although redo surgical aortic valve replacement supported by veno-arterial extracorporeal membrane oxygenation (VA-ECMO) may be considered in patients presenting with cardiogenic shock or cardiac arrest secondary to bioprosthetic valve failure, several factors favoured emergency ViV-TAVI in this case. First, redo cardiac surgery carries substantially higher perioperative morbidity and mortality than first-time surgery (his EuroScore II was 60.2%), particularly in haemodynamically unstable patients requiring urgent intervention. Second, ViV-TAVI can be performed rapidly without the need for repeat sternotomy or cardiopulmonary bypass, reducing procedural time and physiological stress. Therefore, the multidisciplinary heart team judged emergency ViV-TAVI to offer the most appropriate balance of procedural risk and potential benefit for this patient.

Obesity is an important modifier of procedural risk in TAVI. Although some studies suggest an obesity paradox with similar or even improved survival in obese patients, obesity also increases technical difficulty, especially in transfemoral access and closure [3,4]. Evidence on vascular complications is mixed, with some studies showing no overall association with obesity, while other data highlight predictors such as vessel size and sheath diameter [5,6]. The patient's post-procedural groin bleeding, need for endovascular salvage, and later surgical reconstruction are consistent with these known access-related challenges.

Coronary protection is a pre-emptive strategy used during TAVI when there is a risk of coronary obstruction, typically by placing a guidewire with a ready-to-deploy balloon or stent in the coronary artery [7]. It is especially relevant in valve-in-valve procedures, where anatomical factors may increase the likelihood of acute coronary compromise.

Balloon aortic valvuloplasty may provide temporary stabilisation when definitive valve treatment cannot be undertaken immediately [8]. In this case, its use as a bridging strategy prior to definitive valve-in-valve implantation reflects a pragmatic approach in the setting of haemodynamic instability.

Large registry data have demonstrated improving outcomes in TAVI over time, although vascular complications remain clinically significant [9].

This case is especially relevant because the patient presented not merely with symptomatic degeneration but with recurrent pulseless electrical activity arrest, indicating extreme haemodynamic decompensation. Emergency bridging with balloon valvuloplasty followed by definitive valve-in-valve implantation reflects a pragmatic staged strategy used in unstable patients.

This case also underscores the value of multidisciplinary management. Coordination between interventional cardiology, cardiac surgery, intensive care, vascular surgery, and infection services was essential for survival and recovery. The case adds to the literature by illustrating that emergency ViV-TAVI can be performed successfully even after recurrent arrest but that access-site complications may be substantial in patients with high BMI and prior surgical valve replacement.

## Conclusions

Following ViV-TAVI, the patient recovered after 10-15 days in the cardiac intensive care unit and was discharged under close follow-up with cardiology, vascular surgery, and cardiac rehabilitation. At follow-up, he demonstrated significant clinical improvement, with marked resolution of heart failure symptoms and improved exercise tolerance. He reported reduced breathlessness on exertion and no recurrent episodes of acute decompensation. Functionally, he improved to New York Heart Association (NYHA) class II and was able to perform routine daily activities with only mild limitation. Repeat echocardiography demonstrated preserved left ventricular systolic function, a well-seated transcatheter valve with a mean gradient of 20 mmHg and a peak aortic valve velocity of 2.9 m/s, and an estimated pulmonary artery systolic pressure of 40-45 mmHg.

This case underscores that structural degeneration of bioprosthetic valves can present with catastrophic haemodynamic compromise, including recurrent pulseless electrical activity arrest. In such high-risk patients, including those with obesity, ViV-TAVI may provide a life-saving alternative to redo surgery, particularly when combined with early recognition, bridging stabilisation, coronary protection, and coordinated multidisciplinary management.
